# EMPLOYING PHOTOVOICE TO MAP ADOLESCENT LIFE TRAJECTORIES AMID MATERNAL YOUNG-ONSET DEMENTIA: A CASE STUDY

**DOI:** 10.1093/geroni/igad104.3722

**Published:** 2023-12-21

**Authors:** Xueting Tang, Junqiao Wang, Bei Wu, Xiaoyan Cui, Jing Wang

**Affiliations:** Fudan University, Shanghai, Shanghai, China (People’s Republic); Fudan University, Shanghai, Shanghai, China (People’s Republic); New York University, New York City, New York, United States; Fudan University, Shanghai, Shanghai, China (People’s Republic); University of New Hampshire, Durham, New Hampshire, United States

## Abstract

The impact of parental dementia on family dynamics and roles can be substantial, presenting notable challenges for their offspring. Research concerning the life trajectory of adolescents having parents diagnosed with Young-Onset Dementia (YOD) remains limited. This study employs a combination of semi-structured interviews and photovoice through life course lens to delve into the experiences of an adolescent (Alice), exploring how her mother’s condition shapes her life journey and providing novel insights for the development of supportive services. Over a three-month period, Alice captures daily-life moments through photography and selects meaningful photos for sharing. Regular bi-weekly sessions are dedicated to photo-sharing, during which these chosen photos are regarded as pivotal subjects for in-depth exploration in subsequent interview sessions. This approach facilitates gaining insight into her life experience from a nuanced perspective. Thematic analysis reveals four key themes: Firstly, the adaptation of her future ambitions in response to her mother’s condition and increased family responsibilities, resulting in a curtailment of her educational and career prospects; secondly, building a stronger connection with her father and collaborating in shouldering responsibilities. Thirdly, her mother’s prolonged absence during critical phases of personal development impacts her understanding of interpersonal interactions and relationships, prompting a pressing need to establish empathetic connections with others; and lastly, the emergence of Alice’s distinct self-identity and resilience shaped by her unique experiences. These findings underscore the importance of establishing a family-centered support system that addresses the emotional, social, and educational challenges faced by adolescents like Alice as they navigate complex family circumstances.

